# Ketamine modulates disrupted in schizophrenia-1/glycogen synthase kinase-3β interaction

**DOI:** 10.3389/fnmol.2024.1342233

**Published:** 2024-05-22

**Authors:** Jia-Ren Liu, Xiao Hui Han, Koichi Yuki, Sulpicio G. Soriano

**Affiliations:** ^1^Department of Clinical Laboratory, The Fourth Affiliated Hospital of Harbin Medical University, Harbin, China; ^2^Department of Anesthesiology, Perioperative and Pain Medicine, Boston Children’s Hospital, Boston, MA, United States; ^3^Department of Anaesthesia, Harvard Medical School, Boston, MA, United States

**Keywords:** ketamine, lithium, DISC1, GSK-3β, anesthetic neurotoxicity, *N*-methyl-d-aspartate receptor (NMDAR) antagonist

## Abstract

**Introduction:**

Disrupted in schizophrenia-1 (DISC1) is a scaffolding protein whose mutated form has been linked to schizophrenia, bipolar affective disorders, and recurrent major depression. DISC1 regulates multiple signaling pathways involved in neurite outgrowth and cortical development and binds directly to glycogen synthase kinase-3β (GSK-3β). Since ketamine activates GSK-3β, we examined the impact of ketamine on DISC1 and GSK-3β expression.

**Methods:**

Postnatal day 7 rat pups were treated with ketamine with and without the non-specific GSK-3β antagonist, lithium. Cleaved-caspase-3, GSK-3β and DISC1 levels were measured by immunoblots and DISC1 co-localization in neurons by immunofluorescence. Binding of DISC1 to GSK-3β was determined by co-immunoprecipitation. Neurite outgrowth was determined by measuring dendrite and axon length in primary neuronal cell cultures treated with ketamine and lithium.

**Results:**

Ketamine decreased DISC1 in a dose and time-dependent manner. This corresponded to decreases in phosphorylated GSK-3β, which implicates increased GSK-3β activity. Lithium significantly attenuated ketamine-induced decrease in DISC1 levels. Ketamine decreased co-immunoprecipitation of DISC1 with GSK-3β and axonal length.

**Conclusion:**

These findings confirmed that acute administration of ketamine decreases in DISC1 levels and axonal growth. Lithium reversed this effect. This interaction provides a link between DISC1 and ketamine-induced neurodegeneration.

## 1 Introduction

Ketamine is commonly administered for sedation and general anesthesia in infants and children and is associated with neuronal apoptosis, deranged dendritic morphology, and subsequent behavioral deficits in laboratory models of anesthetic-induced neurodegeneration and schizophrenia (Zou et al., 2009; Kruse and Bustillo, 2022). We previously reported that ketamine-induced neuroapoptosis is associated with a temporal increase in glycogen synthase kinase-3β (GSK-3β) activity by reducing phosphorylated GSK-3β (pGSK-3β) (Hur and Zhou, 2010). GSK-3β is a multifunctional kinase that is active in neuronal development and linked to neurodegenerative disorders (Liu J. R. et al., 2013). Given the similarities between ketamine-induced neurotoxicity and neurodegenerative and mood disorders, the role of GSK-3β and its binding partners warranted investigation.

Disrupted in schizophrenia 1 (DISC1) is a GSK-3β binding partner that regulates multiple signaling pathways for diverse processes such as neuronal proliferation, spine regulation and maintenance of synapses (Brandon et al., 2009; Hayashi-Takagi et al., 2010; Ishizuka et al., 2011). DISC1 directly binds to specific phosphorylation sites on domains that decreases GSK-3β activity (Mao et al., 2009; Lipina et al., 2011). A high incidence of schizophrenia and major mood disorders has been linked to an inherited chromosomal translocation of the DISC1 gene in Scottish families (Brandon et al., 2009). Loss of function DISC1 knockout mice have decreased phosphorylated GSK-3β (pGSK-3β), resulting in development of schizophrenia-related behaviors in mice (Lipina et al., 2011). Transient disruption of DISC1 during the neonatal period has been shown to negatively impact synaptic plasticity in mice (Greenhill et al., 2015).

*N*-methyl-d-aspartate receptor (NMDAR) antagonists reduces expression of DISC1 and leads to aberrant migration of newly generated neurons in the hippocampus (Namba et al., 2011) and synaptic spine deterioration (Hayashi-Takagi et al., 2014). These developmental changes are consistent with synaptic pathology seen in laboratory models of both anesthetic-induced neurotoxicity and schizophrenia. This association between NMDAR dysfunction and psychiatric conditions has been established in several laboratory animal and human studies (Snyder and Gao, 2013) and ketamine has been utilized for investigating schizophrenia and mood disorders in preclinical and clinical settings (Suárez Santiago et al., 2023).

Taken together, these observations reveal a potential role of DISC1 and GSK-3β in experimental paradigms of ketamine-induced developmental neurotoxicity and psychiatric disorders. In the present study, we tested the hypothesis that ketamine-induced activation of GSK-3β activity may be due to decreased DISC1 expression. In the present study, measured the effect of ketamine with and without the non-specific GSK-3β inhibitor lithium on DISC1 and GSK-3β expression, neuroapoptosis, and neuronal arborization.

## 2 Materials and methods

### 2.1 Animals and reagents

Pathogen-free Sprague–Dawley (SD) postnatal day 7 (P7) rat pups were obtained from the Charles River Laboratories (Wilmington, MA). All experimental protocols were approved by the Boston Children’s Hospital institutional animal care and use committee (Institute of Laboratory Animal Research Commission on Life Sciences NRC, 1996). Ketamine (Ketalar, Bedford Labs, Bedford, OH) and lithium chloride (Sigma-Aldrich, St. Louis, MO) were obtained from commercial sources.

### 2.2 *In vivo* experiments

The treatments were conducted in a temperature-controlled acrylic container maintained at 36.7°C. As in previous reports, similar conditions resulted in core body temperatures between 36.5 and 37.5°C (Head et al., 2009). Since sex does not affect neuronal death in anesthetic-induced models of developmental neurotoxicity, we utilized both female and male rat pups for the experiments (Lee et al., 2014). The temporal effect of ketamine was determined in P7 rat pups randomly divided into 5 groups (6 rats per group) receiving 4 intraperitoneal (ip) injections of either ketamine (20 mg · kg^−1^) or vehicle (saline 10 mL · kg^−1^) at 90 min intervals over 6 h. Group 1 received 5 consecutive injections of saline (control), group 2: received 2 consecutive doses of ketamine followed by 3 injections of saline, group 3: received 3 consecutive doses of ketamine followed by 2 injections of saline, group 4: received 4 doses of ketamine followed by 1 injection of saline and group 5: received 5 consecutive doses of ketamine. This dosing regimen is similar to that used in previous investigations utilizing a similar experimental paradigm that yields a ketamine plasma concentration of 5.80 ± 3.10 μg^−1^· ml^−1^and brain concentration 2.65 ± 1.60 μg^−1^· g^−1^ (Zou et al., 2009).

To determine the effect of dosage on DISC1 and pGSK-3β (ser 9) levels, a second cohort of rat pups received ketamine (0, 5, 10, or 20 mg·kg^−1^ at 90 min intervals over 6 h, 6 rats per group).

To examine the effect of lithium a non-specific blocker of GSK-3β on DISC1 expression, rat pups were randomly assigned into 4 groups (8 rats per group). The rats received 5 ip injections (10 mL · kg^−1^) of saline, lithium (120 mg · kg^−1^), lithium (120 mg · kg^−1^) and ketamine (20 mg · kg^−1^) or ketamine (20 mg· kg^−1^) alone, at 90 min intervals over 6 h.

The rat pups were kept from their dam and visually monitored for respiratory effort and activity. After the treatment period, the rat pups were euthanized with pentobarbital (100 mg · kg^−1^). The brains from each group were rapidly frozen in liquid nitrogen and processed for protein analysis or immersed in 4% paraformaldehyde and embedded in paraffin for histological processing.

### 2.3 Protein extraction and Western blotting analysis

Protein was extracted from flash-frozen brain tissue with radioimmunoprecipitation assay buffer (Sigma) containing complete (protease and phosphorylase inhibitor cocktail) and 1 mM phenylmethanesulfonyl fluoride. Protein concentrations were measured by BCA protein assay (Bio-Rad, Hercules, CA). The equal amounts of protein were boiled in sodium dodecyl sulfate loading buffer (Bio-Rad), resolved on 8–12% polyacrylamide denaturing (SDS PAGE) gels and transferred to nitrocellulose (Bio-Rad). Antibodies used for Western blotting included rabbit antibodies to cleaved-caspase-3, total and pGSK-3β (ser 9), DISC1 and β-actin (1:2,000, Cell Signaling, Beverly, MA). 12% SDS PAGE gels were utilized to detect DISC1, due to its relatively high molecular weight of DISC1 of 75 kDa. The blots were washed, and the species-matched peroxidase-conjugated secondary antibody was added. Labeled bands from each blot were detected by enhanced chemiluminescence for visualization and quantitation (Thermo Scientific, Waltham, MA). The densities of the specific protein bands were quantified with an image analysis software (Image J 1.42 NIH, United States).

### 2.4 DISC1 immunohistochemistry

Brain sections from the ketamine/lithium/saline-treated cohorts were deparaffinized in xylene and rehydrated through graded alcohol. Endogenous peroxidases were inactivated by immersing the sections in hydrogen peroxide for 10 min, and then were incubated for 10 min with 10% normal goat serum to block non-specific binding. These sections were incubated with anti-DISC1 (Invitrogen) and neuron-specific mouse anti-NeuN (neuronal nuclei antigen) (1:100; Abcam, Cambridge, MA) antibodies overnight, followed by incubation for 90 min with the following secondary antibodies: donkey anti-rabbit cy3-conjugate (1:100, Jackson ImmunoResearch) and Streptavidin-Alexa fluor 488 conjugate (1:100, Life Technologies) antibodies. Both primary and secondary antibodies were diluted in phosphate buffer solution (PBS) containing 0.3% Triton x-100, 0.04% bovine serum albumin, and 0.1% sodium azide. Brain slices were rinsed in PBS before mounting on slides. After mounted sections with 90% glycerol, the slides were imaged with fluorescent microscope (Olympus IX81, Olympus, Japan), and the cell counts under magnification of 400× were performed as described previously (Liu et al., 2012). Three sections from each group were examined and the average number of positive cells was recorded in blinded fashion to the treatment group (6 brain per group).

### 2.5 Structural prediction of DISC1-GSK-3β interaction

DISC1 and GSK-3β amino acid sequences were obtained from National Center for Biotechnology Information (NCBI) database (NP_001158021 and XP_054202359). To predict DISC1 and GSK-3β interaction, we used Alpha Fold 2 software with auto mode without template (Jumper et al., 2021; Mirdita et al., 2022). We used 5 recycles and recycles early stop tolerance 1.0. The predicted structure was analyzed with pyMol.

### 2.6 DISC1/GSK-3β co-immunoprecipitation assay

Protein lysates extracted from the brains of control and ketamine (20 mg· kg^−1^ at 90 min intervals over 6 h cohorts, 6 rats per group) were preincubated with protein A/G agarose beads (Thermo Scientific, Rockford, IL) were washed once with RIPA buffer. Solubilized rat brain tissues (1.0 mg of total protein) were pre-incubated with 20 μL of protein A/G agarose for 2 h at 4°C to reduce non-specific binding. Protein samples were incubated with anti-GSK-3β antibody (1:100, Cell signaling technology, Danvers, MA) overnight and 60 μL of protein A/G agarose was added for 2 h at 4°C. The beads were centrifuged and washed four times in the immunoprecipitation (IP) buffer. Samples were boiled in 2 × SDS sample buffer for 5 min at 95–100°C. Samples were loaded 10% SDS-PAGE gel for western blotting analysis. Approximately 50 μg of protein were used as a positive control. The negative control was prepared using the same protocol with omission of the anti-GSK-3β antibody. Proteins were then transferred to a 0.2 μm nitrocellulose membrane for electrophoresis. Non-specific binding sites were blocked by incubating the blots in 5% non-fat dry milk for 1 h at room temperature and incubated with an appropriate primary, anti-DISC1 (Invitrogen, Carlsbad, CA) and anti-GSK-3β antibodies, at 4°C overnight with a gentle rotation. The blots were incubated with anti-rabbit horseradish peroxidase-linked secondary antibodies and enhanced chemiluminescence substrate. The intensity of each protein band was quantified by densitometry using Image J software.

### 2.7 Imaging of arborization of primary neurons

Given the role of DISC1 on neurite outgrowth, we examine the effect of ketamine on dendritic and axonal morphology in primary neuronal cells from cortices of embryonic day 18 (E18) SD rat fetuses. In brief, embryos were delivered from the dams and euthanized under CO_2_. The cortices of brain were quickly dissected from embryos in ice-cold Hank’s balance salt solution (HBSS) under the dissection microscope (Leica, Buffalo Grove, IL). Cortical neurons (E18) were isolated from cortices after incubation with 0.25% trypsin–EDTA (ethylenediamine-tetraacetic acid) in HBSS for 30 min at 37°C. Cortical neurons were cultured with neurobasal medium (Invitrogen) containing 2% B27 supplement, 500 μM glutamine and 10 μg/mL gentamycin (Invitrogen) in slide chambers coated with poly-d-lysine. After a 3-day incubation period, the E18 cells treated with control solution, lithium 500 μM, ketamine 100 μM, or ketamine 100 μM combined with lithium 500 μM for 6 h. E18 cells were fixed with 1% paraformaldehyde solution and incubated with anti-NeuN and anti-Neurofilament antibodies (EMD Millipore, Billerica, MA) overnight at 4°C to recognize cellular nuclear and axon of neurons, respectively. The slides were incubated with Streptavidin-Alexa fluor 488 conjugate (1:100, Life Technologies) and donkey anti-rabbit cy3-conjugate (1:100, Jackson ImmunoResearch) antibodies for 90 min. The slides were washed with PBS and mounted with soluble 90% glycerol. Five adjacent slides were examined for each of the treatment groups. The cell images were taken under a fluorescent microscope. The length of axons and dendrites were determined by tracing the lengths of the dendrites and axons of each cell by Image J software in blinded fashion to the experimental group.

### 2.8 Statistical analysis

In line with our and other laboratories’ practices, a minimum of six rat pups for each experimental group was utilized to detect a 40% difference in the mean values with 80% power at a significance level of 0.05 (Dell et al., 2002). Changes in cleaved-caspase-3 level were presented as percentage of control value. GSK-3β ratios of the phosphorylated and total forms were calculated. Cleaved-caspase-3 positive cells were presented as absolute values per microscopic field. Data were expressed as mean and standard error of the mean (S.E.M.). Normal distribution of the data was assessed by the Shapiro–Wilk test. The differences of the cleaved-caspase-3, pGSK-3β (ser 9), GSK-3β and DISC1 levels and E18 cell viability were analyzed with one-way ANOVA, followed by a *post hoc* Newman–Keuls multiple comparison test. Since the data from the immunofluorescence assay for cleaved-caspase-3 and NeuN had a skewed distribution, a *Kruskal-Wallis’s* test was applied to analyze differences, followed by Dunn test for multiple comparisons. Data analyses were generated, and plots were constructed using Prism 8 (GraphPad Software, La Jolla, CA).

## 3 Results

### 3.1 Ketamine increased activated caspase-3 and decreased DISC1 and pGSK-3β expression

**Figure 1:**
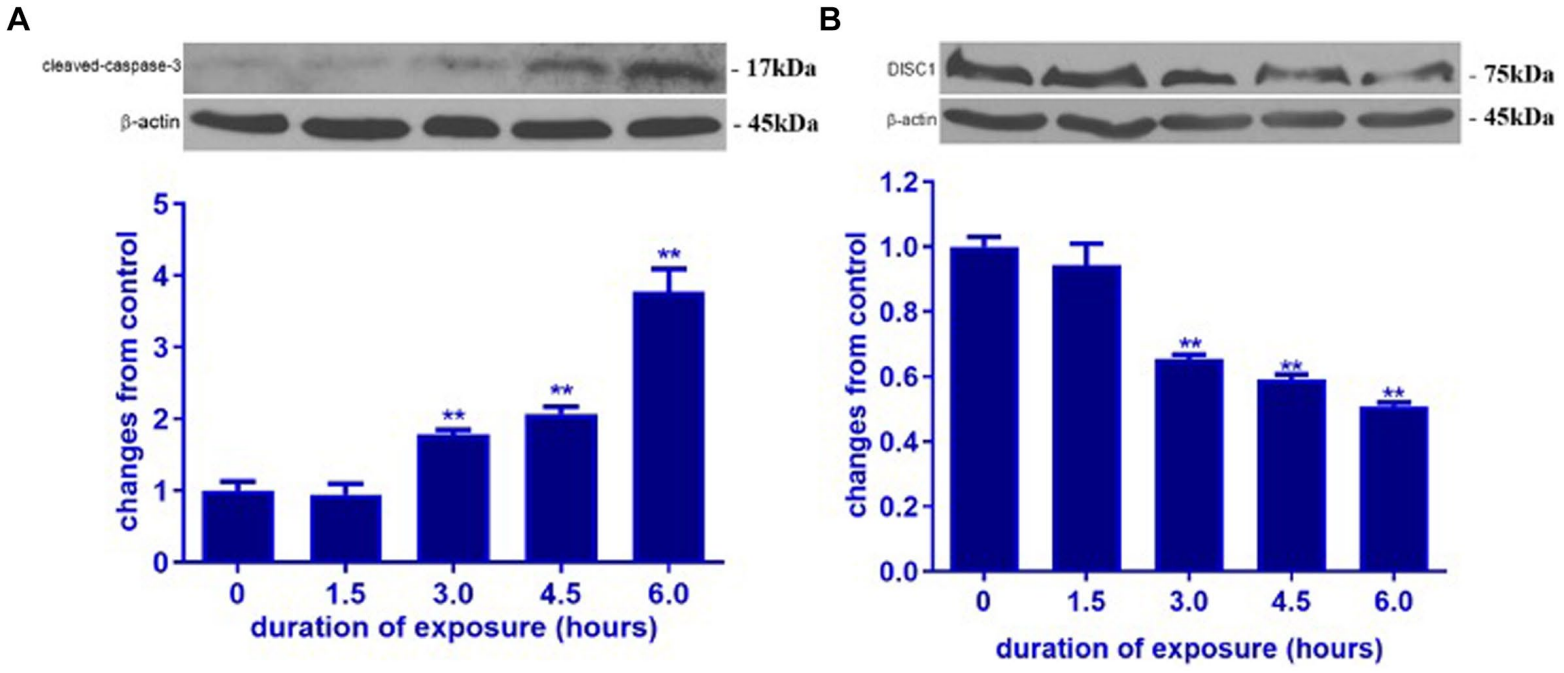
Ketamine increased cleaved-caspase-3 and decreased DISC1 expression in P7 rat brain. Ketamine temporally increased cleaved-caspase-3 protein **(A)** and decreased DISC1 expression **(B)** in P7 rat pups. The data are expressed as the means ± standard deviation (S.D.). ^**^p < 0.01, when compared to the negative control.

**Figure 2:**
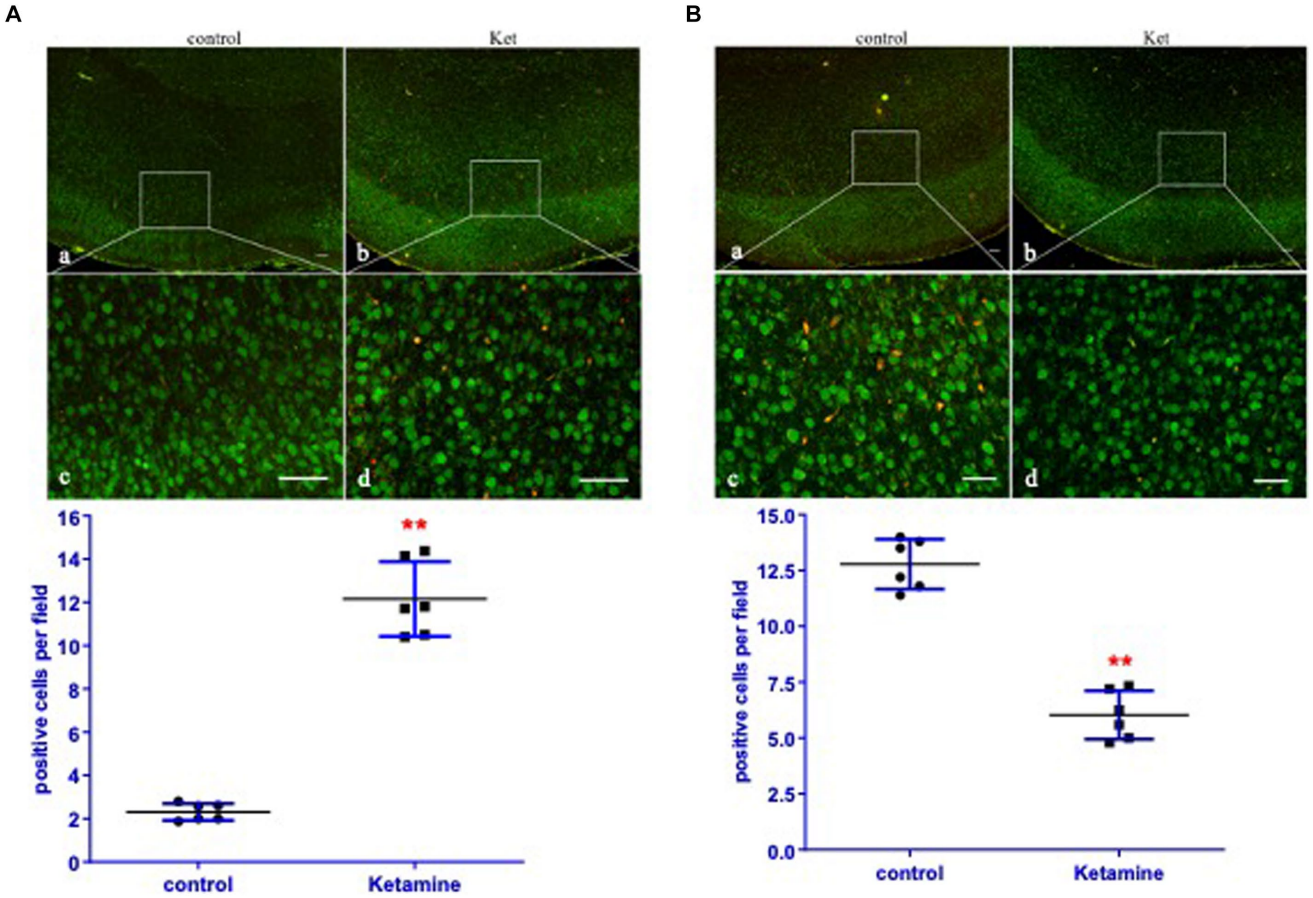
Ketamine increased cleaved-caspase-3 and decreased DISC1 expression primarily in neurons. **(A)** In order to identify the cell-type undergoing apoptosis, the expression of cleaved-caspase-3 (red) and neurons (green, NeuN) in cortices of brain were determined by immunofluorescence. Yellow/orange merged objects identify neurons with cleaved-caspase-3 expression (*n* = 6 rats/group). **(B)** Expression of DISC1 (red) and neurons (green, NeuN) in the cortices of brain tissues were determined by immunofluorescence. Yellow/orange merged images shows neurons with DISC1 expression (*n* = 6 rats/group). The panels are **(a,c)** for the negative control, ketamine panels **(b,d)**. Data are presented as mean ± SEM. Scale bar = **(a,b)** 200 μm, **(c,d)** 25 μm. ^**^p < 0.01, when compared to the control.

**Figure 3:**
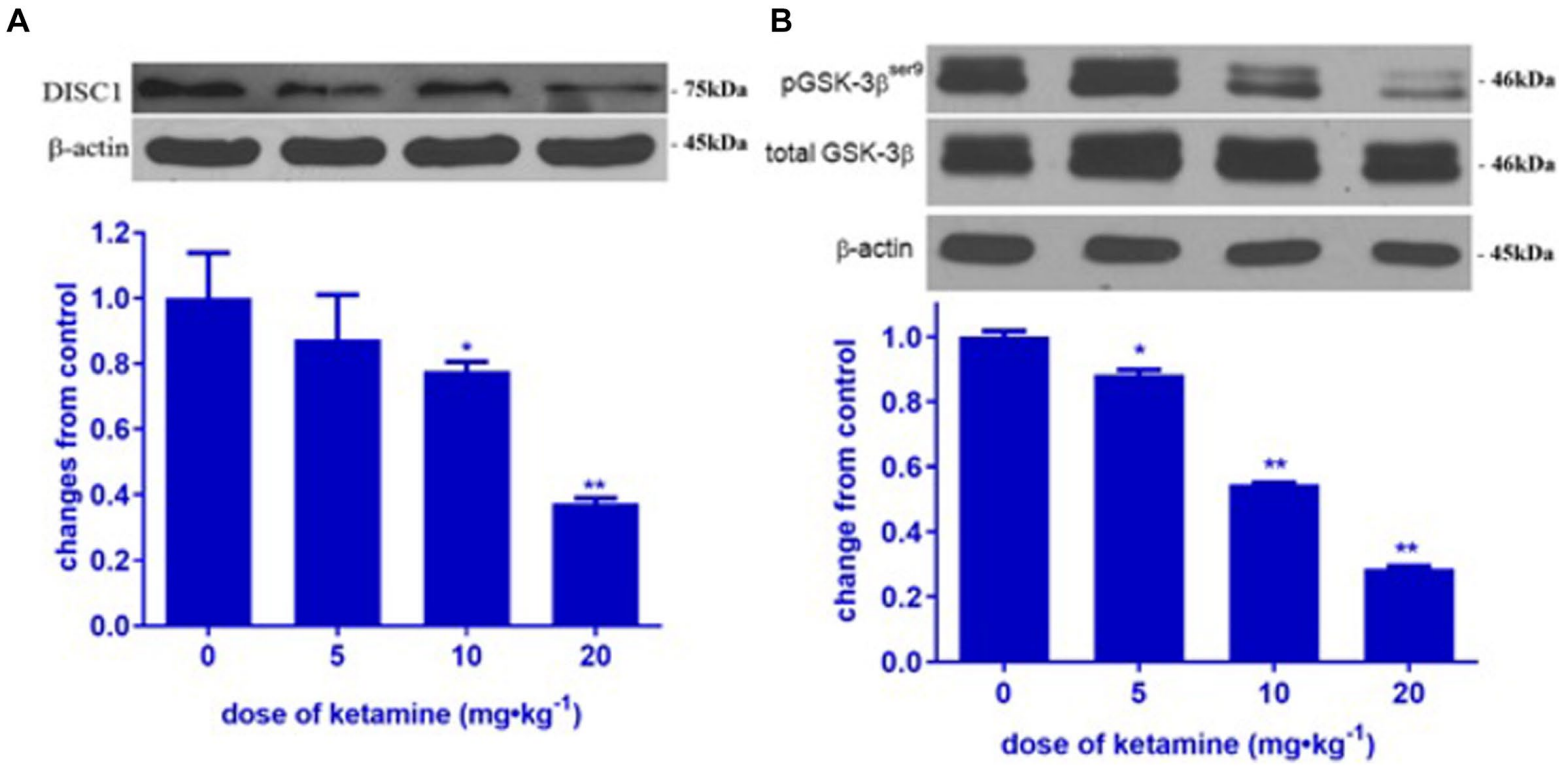
Ketamine decreased DISC1 and pGSK-3β (ser 9) in a dose-dependent manner. Ketamine decreased DISC1 **(A)** and pGSK-3β **(B)** in a dose-dependent manner (*n* = 3 rats/group, ^**^p < 0.01, when compared to control). Data are presented as mean ± SEM.

In order to determine the impact of ketamine neuroapoptosis and DISC1 expression in the brain, the expression of cleaved-caspase-3, pGSK-3β (ser9) and DISC1 was examined by Western blot and immunohistochemistry. Ketamine significantly increased cleaved-caspase-3 and decreased DISC1 expression in p7 brain tissues after 3 h exposure in a time-dependent manner (*p* < 0.05, Figures 1A,B). Immunofluorescence microscopy of brain sections stained with antibodies to cleaved-caspase-3 (red) and NeuN, a neuron-specific nuclear protein (green) antibodies confirmed that ketamine significantly increased apoptosis and decreased DISC1 expression in neurons (Figures 2A,B). Increasing the interval dose of ketamine also significantly decreased DISC1 and pGSK-3β expression (*p* < 0.05 or *p* < 0.01) (Figures 3A,B). The dose dependent reduction of pGSK-3β expression is consistent with our previous report and indicate an increase in GSK-3β activity (Mao et al., 2009).

### 3.2 Lithium attenuated ketamine-induced decreases in DISC1 expression

**Figure 4:**
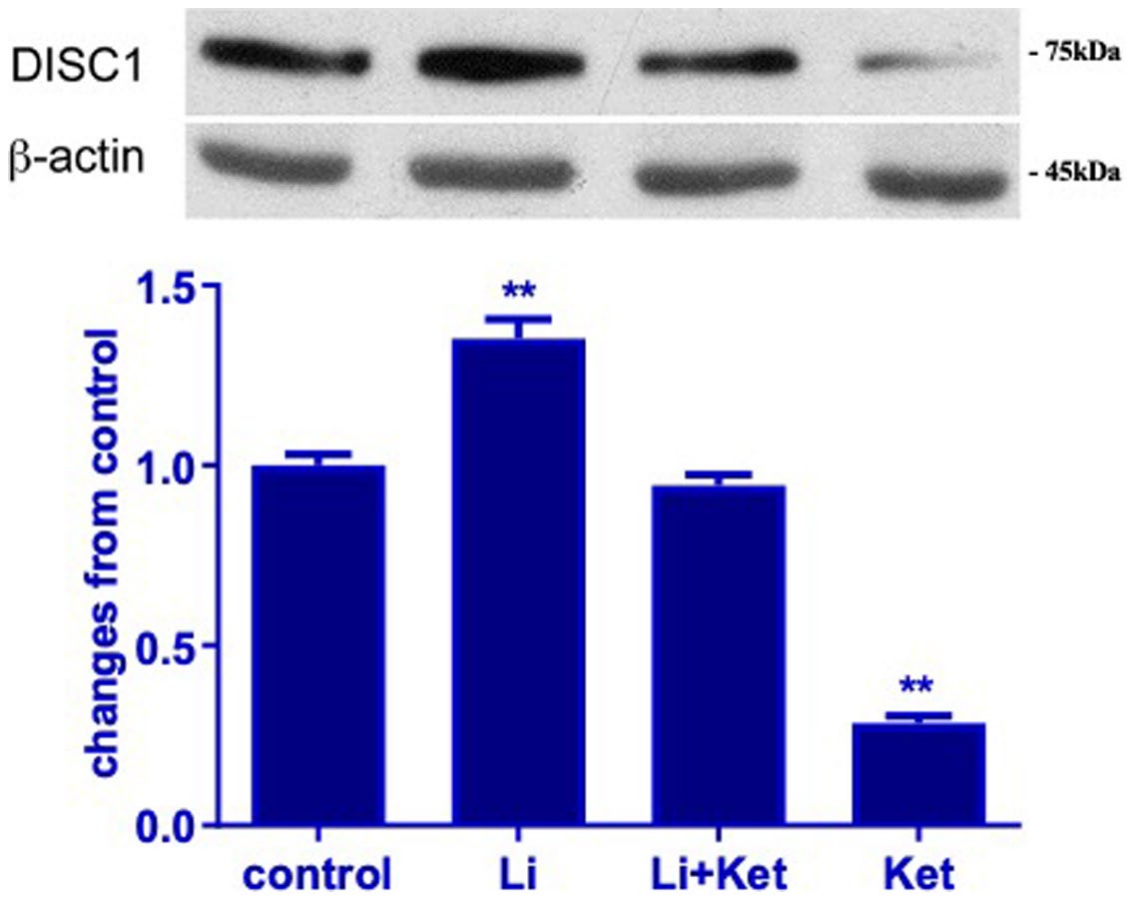
Lithium increased DISC1 expression in naïve rats and mitigated ketamine-induced decreases in DISC1 expression. Western blots from postnatal day 7 brain protein lysates demonstrate that ketamine decreased DISC1 expression. Concurrent administration of lithium and ketamine attenuated this response. Lithium (Li) alone increased DISC1 expression (*n* = 4 rats/group, ^*^p < 0.05, ^**^p < 0.01, when compared to control). Data are presented as mean ± SEM.

To examine the effect of ketamine on the DISC1/GSK-3β binding, lithium, a non-specific inhibitor of GSK-3β, was used to treat P7 rat with or without ketamine. Ketamine at dose of 20 mg · kg^−1^ significantly decreased the expression of DISC1 protein in p7 brain tissues. The addition of lithium significantly attenuated ketamine-induced decreases DISC1 expression (*p* < 0.01) (Figure 4). Interestingly, lithium increased DISC1 protein expression in the absence of ketamine, which suggest that lithium may displace DISC1 from binding sites on GSK-3β.

### 3.3 Predictive modeling of DISC1 and GSK-3β interaction

**Figure 5:**
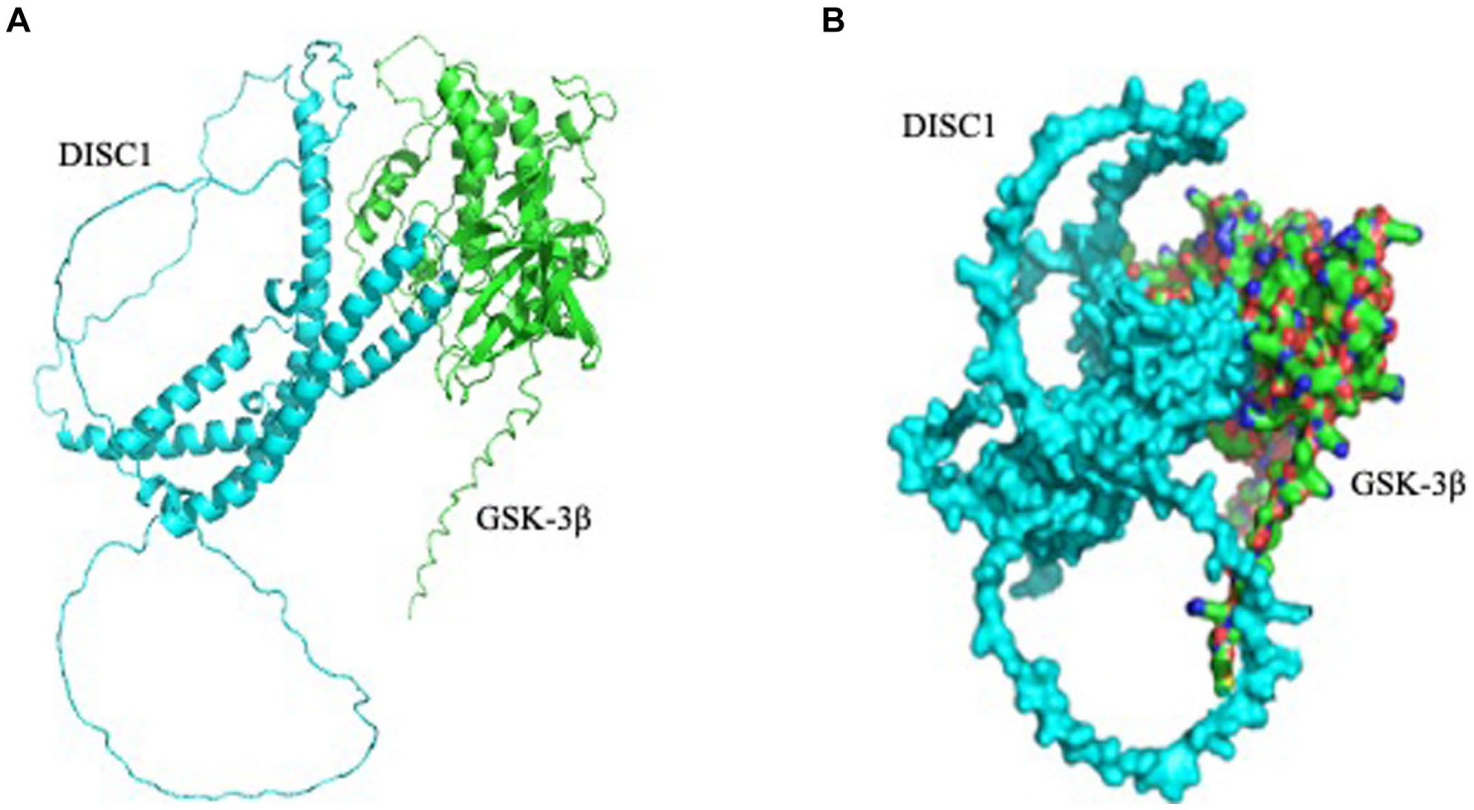
Predicted interaction of DISC1 (cyan) and GSK-3β (green). **(A)** DISC1 and GSK-3β were shown in cartoon mode. **(B)** DISC1 and GSK-3β complex wereshown in surface mode.

Based on the several reports, we hypothesized that DISC1 and GSK-3β would directly bind to each other (Mao et al., 2009; Lipina et al., 2011). Using AlphaFold2, we predicted structural relationship between DISC1 and GSK-3β. Our docking suggested DISC1 structurally interacted with GSK-3β (Figure 5).

### 3.4 Ketamine decreased DISC1 binding to GSK-3β

**Figure 6:**
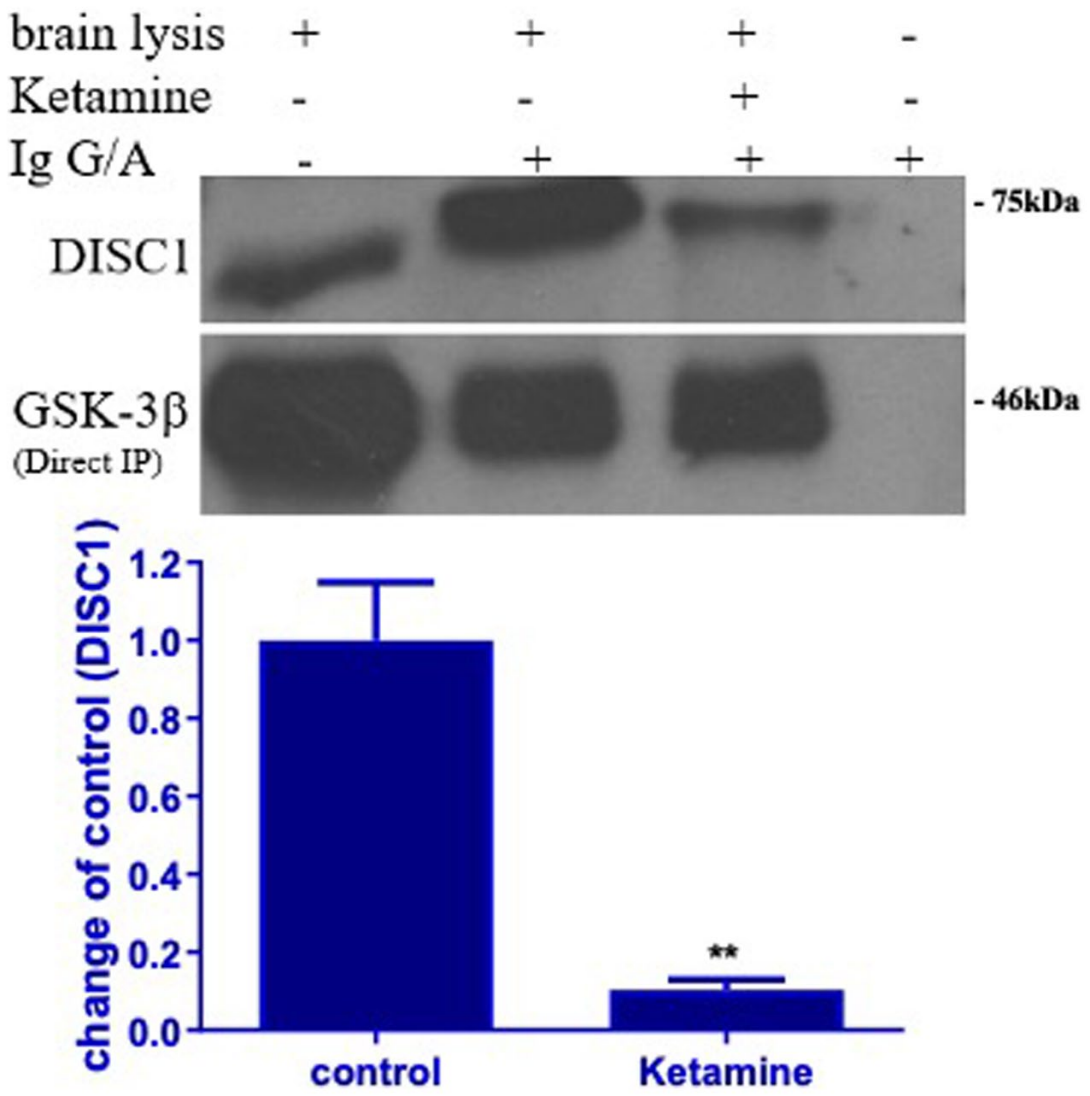
Ketamine decreases DISC1 binding to GSK-3β. Binding of DISC1 to GSK-3 β was determined in brain tissues by co-immunoprecipitation (Co-IP). Ketamine significantly decreased the expression of free DISC1 protein, indicating that ketamine (Ket) decreased GSK-3β binding to DISC1 protein (*n* = 3 rats/group, ^**^p < 0.01, when compared to control). Data are presented as mean ± SEM.

DISC1 has been shown to regulate GSK-3 β activity by directly binding to multiple domains on GSK-3β. To determine the effect of ketamine on DISC1-GSK-3β binding, protein lysates extracted from the brains of control and ketamine treated rats were subjected to co-immunoprecipitation assay. Ketamine induced a 40 and 60% reduction in absolute DISC1 expression standard western blots (Figures 1B, 3A). The experiment yielded a 90% reduction in bound DISC1 (Figure 6), which suggest a reduction in bound DISC1, which is lower than predicted by the standard DISC1 immunoblots. Therefore, the structural prediction of DISC1-GSK-3β interaction using Alpha Fold 2 software corroborates with our co-immunoprecipitation results.

### 3.5 Ketamine attenuated growth of dendrites and axons in primary neurons and lithium mitigated this response

**Figure 7:**
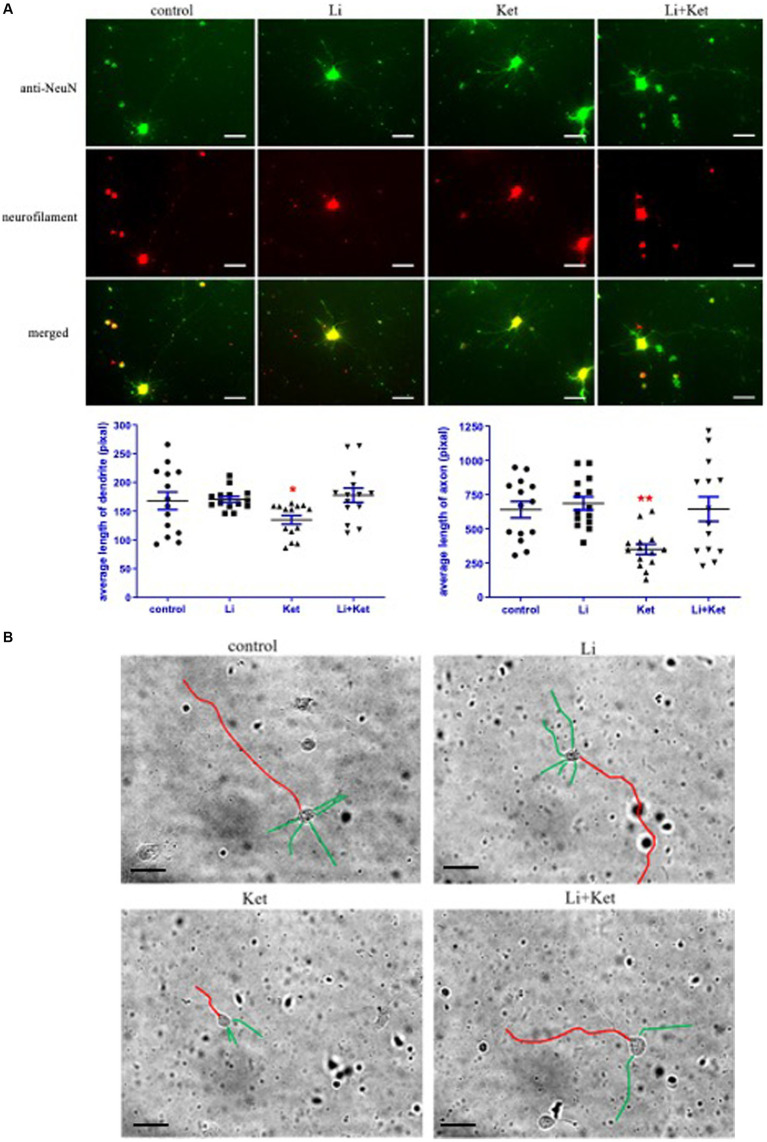
Ketamine decreased the length of dendrites and axons in E18 cells and lithium attenuated this response. **(A)** E18 cells treated with control solution, lithium (Li), ketamine (Ket), or ketamine combined with lithium for 6 h. Cells were fixed and stained with anti-NeuN (green) and Neurofilament (red) antibodies. The individual and merged images were taken by a fluorescence microscope. Scale bar = 25 μm. ^*^p < 0.05, ^**^p < 0.01, when compared to control. **(B)** Traces of the lengths of the dendrites (green) and axons (red) were measured on greyscale images of the E18 cells using Image J software. Scale bar = 50 μm.

DISC1 is an important regulator of dendrite formation and function, and axonal extension (Miyoshi et al., 2003). To examine dendrite formation and axonal extension in this experimental paradigm, E18 cells were treated ketamine with or without lithium for 6 h. Ketamine decreased dendritic and axonal length of E18 cells (*p* < 0.05) (Figure 7). Lithium, a non-specific inhibitor of GSK-3β, significantly mitigated the effects of ketamine on morphological changes in E18 cells. In addition, lithium itself did not change the morphology of E18 cells. Taken together, these results suggest that ketamine-induced reduction of dendritic and axonal growth are modulated by GSK-3β-DISC1 binding.

## 4 Discussion

This study demonstrates that ketamine induced a dose-and duration-dependent decrease in DISC1 expression and phosphorylation of GSK-3β, resulting increased neuroapoptosis and attenuated growth of dendrites and axons. Ketamine also decreased DISC1 binding to GSK-3β. These findings mirror published reports on aberrant DISC1 expression models in neuronal development and psychiatric disorders (Brandon and Sawa, 2011). Furthermore, lithium, a non-specific inhibitor of GSK-3β, normalized the levels of DISC1 to control levels, attenuated ketamine-induced neuroapoptosis, and preserved dendritic arborization.

Since ketamine decreases the level of DISC1 protein, we determined if it affects DISC1 binding partner, GSK-3β, a cytoplasmic serine/threonine protein kinase involved in signaling pathways that modulate cell survival and development. In this study, ketamine significantly decreased the expression of phosphorylated GSK-3β (ser 9) protein in p7 brain tissues in a dose-dependent manner (Figure 3B), indicating that ketamine increases GSK-3β activity in the developing brain. Activation of GSK-3β is associated with psychiatric disorders (Lovestone et al., 2007; Rowe et al., 2007). Lithium is a non-specific inhibitor of GSK-3β widely used to treat psychiatric and mood disorders and also protects against anesthetic-induced neurotoxicity (Noguchi et al., 2016). Lithium specifically attenuated neuroapoptosis induced by ketamine, which may be due to increased GSK-3β activity (Liu J. R. et al., 2013).

Interestingly, in the present study, lithium not only attenuated ketamine-decreases DISC1 protein level, but it also significantly increased the level of DISC1 protein in naïve P7 brain tissues. Lithium itself increases pGSK-3β at ser 9 (Kirshenboim et al., 2004). Functionally, GSK-3β directly binds to the N terminus of DISC1 and is inactivated by direct phosphorylation by DISC1 (Mao et al., 2009). In the present study, our findings showed that ketamine disassociated GSK-3β binding to DISC1, which in turn may increase GSK-3β activity (Figure 6).

DISC1 and its downstream partners play a fundamental role in neurite development (Miyoshi et al., 2003). To determine if ketamine affects dendritic morphology, primary neuronal cells isolated from day 18 embryos of SD rats were treated with ketamine and lithium. The results showed that ketamine significantly decreased the length of dendrites and axons, and that lithium significantly mitigated this effect in E18 cells (Figure 7). Thus, ketamine affects neurite development of neurons. Blocking GSK-3β by lithium can attenuate the detrimental impact of ketamine on the neuronal morphology. Our current findings demonstrate that lithium not only mitigates neuroapoptosis induced by ketamine but also maintains DISC1 expression. These observations suggest that a therapeutic target for lithium in the management of ketamine-induced neurotoxicity as well as psychiatric disorders.

Another potential parallel pathway in ketamine-induced neurodegeneration is through upregulation of the NMDAR (Liu F. et al., 2013). DISC1 knockdown leads to increased expression of NMDAR subunit and aberrant currents (Wei et al., 2014), which is similar to ketamine-induced upregulation of the NMDAR expression leading increase influx of calcium (Wang et al., 2017). Thus, DISC1 has a pivotal role on the regulation of NMDAR function.

There are several limitations to our experimental approach. DISC1 and GSK-3β are hub proteins that involved in multiple parallel biological pathways. In this report we expanded our previous observation that ketamine modulates phosphorylation of GSK-3β as a potential mechanism for neuroapoptosis in P7 rat pups in an experimental model of anesthetic-induced neurotoxicity. However, GSK-3β regulates multiple neurodevelopmental processes beyond the scope of this investigation. Since DISC1 and GSK-3β are constitutively expressed, the current report did not examine the effect of overexpression or knockdown of these proteins on ketamine-induced neurodegeneration. Furthermore, lithium modulates DISC1 and GSK-3β function, it has multiple molecular targets which may involve additional intracellular pathways in this experimental paradigm. Future investigation should consider the use of gene knockout or overexpression in transgenic mice and primary neuronal cell cultures for more specificity. Since relatively large doses typically are utilized in experimental paradigms of anesthetic-induced neurotoxicity (Hayashi et al., 2002; Zou et al., 2009; Liu et al., 2012; Liu J. R. et al., 2013), our report does not address the clinically relevant use of low dose ketamine in the treatment of major depression (Nunez et al., 2023). Finally, our findings are limited to the impact of acute exposure to ketamine in P7 rat pups. Future investigations should also explore the changes in DISC1 expression in at fetal and later developmental stages and the effect of both lower doses and chronic administration of ketamine on DISC1/GSK-3β interactions.

In summary, we demonstrate that acute exposure to ketamine decreases DISC1 expression in the developing rat brain. This corresponded to decreases in phosphorylated GSK-3β, which denotes increased GSK-3β activity. Ketamine combined with lithium not only attenuated neuroapoptosis, but also maintained DISC1 levels. Given the potential for ketamine-induced neurotoxicity in pediatric patients recieving sedation and general anesthesia, our findings demonstrate similar aberrant DISC1-GSK-3β interactions that occur in neurodegenerative and psychiatric disorders.

## Data availability statement

The raw data supporting the conclusions of this article will be made available by the authors, without undue reservation.

## Ethics statement

The animal study was approved by Boston Children’s Hospital institutional animal care and use committee. The study was conducted in accordance with the local legislation and institutional requirements.

## Author contributions

J-RL: Conceptualization, Data curation, Formal analysis, Investigation, Methodology, Project administration, Supervision, Validation, Visualization, Writing – original draft, Writing – review & editing. XH: Data curation, Formal analysis, Investigation, Methodology, Writing – review & editing. KY: Conceptualization, Data curation, Formal analysis, Investigation, Supervision, Writing – review & editing. SS: Conceptualization, Data curation, Formal analysis, Funding acquisition, Investigation, Methodology, Project administration, Resources, Supervision, Validation, Visualization, Writing – original draft, Writing – review & editing.

## References

[ref1] Brandon N. J. Millar J. K. Korth C. Sive H. Singh K. K. Sawa A. (2009). Understanding the role of DISC1 in psychiatric disease and during normal development. J. Neurosci. 29, 12768–12775. doi: 10.1523/JNEUROSCI.3355-09.2009, PMID: 19828788 PMC6665304

[ref2] Brandon N. J. Sawa A. (2011). Linking neurodevelopmental and synaptic theories of mental illness through DISC1. Nat. Rev. Neurosci. 12, 707–722. doi: 10.1038/nrn3120, PMID: 22095064 PMC3954824

[ref3] Dell R. B. Holleran S. Ramakrishnan R. (2002). Sample size determination. ILAR J. 43, 207–213. doi: 10.1093/ilar.43.4.207, PMID: 12391396 PMC3275906

[ref4] Greenhill S. D. Juczewski K. de Haan A. M. Seaton G. Fox K. Hardingham N. R. (2015). Adult cortical plasticity depends on an early postnatal critical period. Science 349, 424–427. doi: 10.1126/science.aaa8481, PMID: 26206934

[ref5] Hayashi H. Dikkes P. Soriano S. G. (2002). Repeated administration of ketamine may lead to neuronal degeneration in the developing rat brain. Paediatr. Anaesth. 12, 770–774. doi: 10.1046/j.1460-9592.2002.00883.x, PMID: 12519135

[ref6] Hayashi-Takagi A. Araki Y. Nakamura M. Vollrath B. Duron S. G. Yan Z.. (2014). PAKs inhibitors ameliorate schizophrenia-associated dendritic spine deterioration in vitro and in vivo during late adolescence. Proc. Natl. Acad. Sci. USA 111, 6461–6466. doi: 10.1073/pnas.1321109111, PMID: 24706880 PMC4035976

[ref7] Hayashi-Takagi A. Takaki M. Graziane N. Seshadri S. Murdoch H. Dunlop A. J.. (2010). Disrupted-in-schizophrenia 1 (DISC1) regulates spines of the glutamate synapse via Rac1. Nat. Neurosci. 13, 327–332. doi: 10.1038/nn.2487, PMID: 20139976 PMC2846623

[ref8] Head B. P. Patel H. H. Niesman I. R. Drummond J. C. Roth D. M. Patel P. M. (2009). Inhibition of p75 neurotrophin receptor attenuates isoflurane-mediated neuronal apoptosis in the neonatal central nervous system. Anesthesiology 110, 813–825. doi: 10.1097/ALN.0b013e31819b602b, PMID: 19293698 PMC2767332

[ref9] Hur E.-M. Zhou F.-Q. (2010). GSK3 signalling in neural development. Nat. Rev. Neurosci. 11, 539–551. doi: 10.1038/nrn2870, PMID: 20648061 PMC3533361

[ref10] Institute of Laboratory Animal Research Commission on Life Sciences NRC (1996). Guide for the care and use of laboratory animals. Washington, D.C.: The National Academy Press.

[ref11] Ishizuka K. Kamiya A. Oh E. C. Kanki H. Seshadri S. Robinson J. F.. (2011). DISC1-dependent switch from progenitor proliferation to migration in the developing cortex. Nature 473, 92–96. doi: 10.1038/nature09859, PMID: 21471969 PMC3088774

[ref12] Jumper J. Evans R. Pritzel A. Green T. Figurnov M. Ronneberger O.. (2021). Highly accurate protein structure prediction with AlphaFold. Nature 596, 583–589. doi: 10.1038/s41586-021-03819-2, PMID: 34265844 PMC8371605

[ref13] Kirshenboim N. Plotkin B. Shlomo S. B. Kaidanovich-Beilin O. Eldar-Finkelman H. (2004). Lithium-mediated phosphorylation of glycogen synthase kinase-3beta involves PI3 kinase-dependent activation of protein kinase C-alpha. J. Mol. Neurosci. 24, 237–246. doi: 10.1385/JMN:24:2:237, PMID: 15456937

[ref14] Kruse A. O. Bustillo J. R. (2022). Glutamatergic dysfunction in schizophrenia. Transl. Psychiatry 12:500. doi: 10.1038/s41398-022-02253-w 36463316 PMC9719533

[ref15] Lee B. H. Chan J. T. Kraeva E. Peterson K. Sall J. W. (2014). Isoflurane exposure in newborn rats induces long-term cognitive dysfunction in males but not females. Neuropharmacology 83, 9–17. doi: 10.1016/j.neuropharm.2014.03.011, PMID: 24704083 PMC4077337

[ref16] Lipina T. V. Kaidanovich-Beilin O. Patel S. Wang M. Clapcote S. J. Liu F.. (2011). Genetic and pharmacological evidence for schizophrenia-related Disc1 interaction with GSK-3. Synapse 65, 234–248. doi: 10.1002/syn.20839, PMID: 20687111 PMC4485461

[ref17] Liu J. R. Baek C. Han X. H. Shoureshi P. Soriano S. G. (2013). Role of glycogen synthase kinase-3β in ketamine-induced developmental neuroapoptosis in rats. Br. J. Anaesth. 110, i3–i9. doi: 10.1093/bja/aet057, PMID: 23533250

[ref18] Liu J. R. Liu Q. Li J. Baek C. Han X. H. Athiraman U.. (2012). Noxious stimulation attenuates ketamine-induced neuroapoptosis in the developing rat brain. Anesthesiology 117, 64–71. doi: 10.1097/ALN.0b013e31825ae693 22617253

[ref19] Liu F. Patterson T. A. Sadovova N. Zhang X. Liu S. Zou X.. (2013). Ketamine-induced neuronal damage and altered N-methyl-D-aspartate receptor function in rat primary forebrain culture. Toxicol. Sci. 131, 548–557. doi: 10.1093/toxsci/kfs296, PMID: 23065140 PMC3551423

[ref20] Lovestone S. Killick R. Di Forti M. Murray R. (2007). Schizophrenia as a GSK-3 dysregulation disorder. Trends Neurosci. 30, 142–149. doi: 10.1016/j.tins.2007.02.002, PMID: 17324475

[ref21] Mao Y. Ge X. Frank C. L. Madison J. M. Koehler A. N. Doud M. K.. (2009). Disrupted in schizophrenia 1 regulates neuronal progenitor proliferation via modulation of GSK3beta/beta-catenin signaling. Cell 136, 1017–1031. doi: 10.1016/j.cell.2008.12.044, PMID: 19303846 PMC2704382

[ref22] Mirdita M. Schütze K. Moriwaki Y. Heo L. Ovchinnikov S. Steinegger M. (2022). ColabFold: making protein folding accessible to all. Nat. Methods 19, 679–682. doi: 10.1038/s41592-022-01488-1, PMID: 35637307 PMC9184281

[ref23] Miyoshi K. Honda A. Baba K. Taniguchi M. Oono K. Fujita T.. (2003). Disrupted-in-schizophrenia 1, a candidate gene for schizophrenia, participates in neurite outgrowth. Mol. Psychiatry 8, 685–694. doi: 10.1038/sj.mp.4001352, PMID: 12874605

[ref24] Namba T. Ming G.-L. Song H. Waga C. Enomoto A. Kaibuchi K.. (2011). NMDA receptor regulates migration of newly generated neurons in the adult hippocampus via disrupted-in-schizophrenia 1 (DISC1). J. Neurochem. 118, 34–44. doi: 10.1111/j.1471-4159.2011.07282.x, PMID: 21517847 PMC4142346

[ref25] Noguchi K. K. Johnson S. A. Kristich L. E. Martin L. D. Dissen G. A. Olsen E. A.. (2016). Lithium protects against anaesthesia neurotoxicity in the infant primate brain. Sci. Rep. 6:22427. doi: 10.1038/srep22427, PMID: 26951756 PMC4782073

[ref26] Nunez N. A. Joseph B. Kumar R. Douka I. Miola A. Prokop L. J.. (2023). An update on the efficacy of single and serial intravenous ketamine infusions and esketamine for bipolar depression: a systematic review and meta-analysis. Brain Sci. 13:1672. doi: 10.3390/brainsci13121672, PMID: 38137120 PMC10741553

[ref27] Rowe M. K. Wiest C. Chuang D. M. (2007). GSK-3 is a viable potential target for therapeutic intervention in bipolar disorder. Neurosci. Biobehav. Rev. 31, 920–931. doi: 10.1016/j.neubiorev.2007.03.002, PMID: 17499358 PMC2020444

[ref28] Snyder M. A. Gao W. J. (2013). NMDA hypofunction as a convergence point for progression and symptoms of schizophrenia. Front. Cell. Neurosci. 7:31. doi: 10.3389/fncel.2013.00031 23543703 PMC3608949

[ref29] Suárez Santiago J. E. Roldán G. R. Picazo O. (2023). Ketamine as a pharmacological tool for the preclinical study of memory deficit in schizophrenia. Behav. Pharmacol. 34, 80–91. doi: 10.1097/FBP.0000000000000689, PMID: 36094064

[ref30] Wang C. Liu F. Patterson T. A. Paule M. G. Slikker W. Jr. (2017). Relationship between ketamine-induced developmental neurotoxicity and NMDA receptor-mediated calcium influx in neural stem cell-derived neurons. Neurotoxicology 60, 254–259. doi: 10.1016/j.neuro.2016.04.015, PMID: 27132109

[ref31] Wei J. Graziane N. M. Wang H. Zhong P. Wang Q. Liu W.. (2014). Regulation of N-methyl-D-aspartate receptors by disrupted-in-schizophrenia-1. Biol. Psychiatry 75, 414–424. doi: 10.1016/j.biopsych.2013.06.009, PMID: 23906531 PMC3864617

[ref32] Zou X. Patterson T. A. Sadovova N. Twaddle N. C. Doerge D. R. Zhang X.. (2009). Potential neurotoxicity of ketamine in the developing rat brain. Toxicol. Sci. 108, 149–158. doi: 10.1093/toxsci/kfn270, PMID: 19126600 PMC2721655

